# Ecophysiological roles of abaxial anthocyanins in a perennial understorey herb from temperate deciduous forests

**DOI:** 10.1093/aobpla/plv042

**Published:** 2015-04-28

**Authors:** Beatriz Fernández-Marín, Raquel Esteban, Fátima Míguez, Unai Artetxe, Verónica Castañeda, Marta Pintó-Marijuan, José María Becerril, José Ignacio García-Plazaola

**Affiliations:** 1 Department of Plant Biology and Ecology, University of the Basque Country (UPV/EHU), Apdo. 644, E-48080 Bilbao, Spain; 2 IdAB-CSIC-UPNA-Government of Navarre, E-31192 Pamplona, Spain; 3 UPNA, E-31192 Pamplona, Spain; 4 Department of Plant Biology, University of Barcelona. Av. Diagonal 643, E-08028 Barcelona, Spain; 5 Present address: Institute of Botany and Center for Molecular Biosciences Innsbruck, University of Innsbruck, Sterwartestraße 15, A-6020 Innsbruck, Austria

**Keywords:** Abaxial anthocyanins, lactucaxanthin, photoprotection, *Saxifraga hirsuta*, spectrum, winter

## Abstract

Accumulation of abaxial anthocyanins is an intriguing leaf trait particularly common among deeply shaded understorey plants of tropical and temperate forests whose ecological significance is still not properly understood. To shed light on it, possible ecophysiological roles of abaxial anthocyanins were tested in the perennial understorey herb of temperate deciduous forests *Saxifraga hirsuta*, chosen as a model species due to the coexistence of green and anthocyanic leaves and the presence of an easily removable lower anthocyanic epidermis. Anthocyanins accumulated during autumn, which temporally matched the overstorey leaf fall. Patterns of development of abaxial anthocyanins and direct measurements of photochemical efficiency under monochromatic light were not consistent with a photoprotective hypothesis. Enhancement of light capture also seemed unlikely since the back-scattering of red light towards the lower mesophyll was negligible. Seed germination was similar under acyanic and anthocyanic leaves. A relevant consequence of abaxial anthocyanins was the dramatic reduction of light transmission through the leaf. The dark environment generated underneath the *Saxifraga* canopy was enhanced by the horizontal repositioning of leaves, which occurs in parallel with reddening. This might play a role in biotic interactions by inhibiting vital processes of competitors, which may be of especial importance in spring before the overstorey leaves sprout.

## Abstract (precis)

Red pigmentation in the lower surface of leaves is a common phenomenon in herb species growing in temperate and tropical forests. Nevertheless, its function is still not completely understood. We studied this process of reddening in the leaves of *Saxifraga hirsuta* living in a beech forest, to establish its relation with environmental factors and its potential function. We observed that the reddening occurs during autumn and that it strongly reduces the amount of light that can pass through the leaf. The dark environment generated underneath might play a role in the biotic interactions by inhibiting vital processes of competitors.

## Introduction

Anthocyanins are pigmented flavonoids responsible for most of the red, pink, purple and blue colours observed in plant tissues, including most of the cases of red leaf colouration in angiosperms (Manetas 2006; Hughes 2011). In leaves, they can be located in the upper and/or lower epidermis, in the palisade and/or spongy mesophyll or in any combination of those leaf cell layers (Lee and Collins 2001). Anthocyanins, as glucosides of anthocyanidins, are water-soluble molecules that usually accumulate in vacuoles and are possibly ubiquitous in green leaves (at low quantities that cannot mask the chlorophyll colour) (Manetas 2006). Nevertheless, under certain circumstances anthocyanin over-production is manifested as leaf redness, e.g. leaf senescence, wounding, pathogen attack, leaf development, nutrient deficiency, UV-B, low temperatures and constitutively in some specific taxa (reviewed in Manetas 2006).

Classically, it has been assumed that leaf anthocyanins may have an important photoprotective role since they show antioxidant and sunscreen functions. Anthocyanins are powerful scavengers of reactive oxygen species (ROS) *in vitro*. Nonetheless, experimental evidence for their *in vivo* role in the antioxidant defence system is controversial, scarce and sparse (for a review, see Kytridis and Manetas 2006; Manetas 2006). Because of their location within cells (in the vacuole and not in the chloroplast, which is the main site of ROS production) and their co-accumulation with other phenolics and flavonoids that may obscure their functions, anthocyanins alone are difficultly related to the antioxidant capacities of red leaves (Manetas 2006). Some ROS however, such as H_2_O_2,_ can enter the vacuole through aquaporins and be neutralized by anthocyanins (Bienert *et al*. 2006). When they are located in the adaxial (upper) surface of leaves, it has been demonstrated that anthocyanins can act as light attenuators and protect underlying cell layers from photoinhibition, through the absorption of blue-green light (Chalker-Scott 1999; Gould *et al*. 2002; Gould 2004). In contrast, this function in light attenuation has not been supported in several species (Woodall *et al*. 1998; Esteban *et al*. 2008; Nikiforou and Manetas 2010) in which anthocyanic tissues are more sensitive to photoinhibition than green ones. Nevertheless, in some of these cases, the confounding effects of lower leaf nitrogen or chlorophyll content in anthocyanic individuals may have obscured the photoprotective effects of anthocyanins. This proposed ‘sunscreen’ function of the anthocyanins becomes even weaker when they are accumulated in the abaxial (lower) part of the leaf.

Anthocyanins located in the abaxial (lower) surface of leaves are common in understorey plants, especially of the tropics (Lee and Collins 2001). Despite the widespread distribution of abaxial reddening among understorey taxa, very little is known about its ecophysiological function (Hughes *et al*. 2008). Two main hypotheses have been suggested. Lee *et al*. (1979) proposed the ‘back-scattering’ hypothesis, which proposes that anthocyanins close to the lower epidermis may reflect adaxially transmitted red light back into the mesophyll, to maximize the absorption of red photons by the mesophyll cells, which could be especially advantageous in light-limited environments. On the other hand, more recently, some authors have proposed an alternative/opposite theory in which abaxial anthocyanins would contribute to attenuate the internal scattering of green light, protecting photosynthetic mesophyll cells during sunflecks and particularly during sun patches, which are longer in duration (Hughes *et al*. 2008, 2014).

Although a series of other more ecological (i.e. anti-herbivore, reviewed in Manetas 2006) hypotheses have also been postulated, the function of abaxial anthocyanins is still a matter of debate. To illuminate this remarkable but still not properly understood phenomenon, *Saxifraga hirsuta* was used as a model species. This is a perennial herb that usually grows over limestone in the shady and humid understorey of temperate forests of the Northern hemisphere. Due to the temporary coexistence of completely green (acyanic) and abaxially red (anthocyanic) leaves within the same plant, and the presence of an easily removable lower epidermis, *S. hirsuta* is a very suitable model to study the role of abaxial anthocyanins in understorey species. Taking advantage of these properties, we aimed (i) to characterize the process of abaxial reddening of *S. hirsuta* leaves in relation with environmental abiotic factors, (ii) to determine the effect that abaxial anthocyanins may induce in the vertical transmission profile of light through the leaf and (iii) to check for photoprotective and alternative functions of foliar abaxial anthocyanins.

## Methods

### Plant material, sampling and site of the study

Plant material was obtained from a natural population of *S. hirsuta* growing over exposed limestone with a thin layer of litter (dead leaves) in the understorey of a beech (*Fagus sylvatica*) forest. The experimental site was located in Monte Santiago Natural Monument (Burgos, northern Spain, 42°56′N, 3°00′W, 900 m above sea level (a.s.l.)). The site is characterized by a temperate oceanic climate, with annual rainfall 1116 mm and mean temperatures ranging from 16 °C in August to 2.7 °C in February. Climatic reference data for a place sited out of the forest were obtained for the nearest meteorological station (Orduña, Basque Agency of Meteorology, Euskalmet) located in the vicinity of Monte Santiago at 4 km from the study site and 934 m a.s.l. Additionally, to characterize the microclimate of the habitat of *S. hirsuta*, an automatic weather station (MiniCube VV/VX16, EMS, Brno, CZ) was installed in the understorey of the study site, in a place representative of that preferred by this species. Data loggers were programmed to record 1h averages of environmental measurements taken every minute from August 2012 to May 2013. For experiments performed in the laboratory, leaves were excised from the plants and preserved in darkness at saturating relative humidity (RH). Fresh material was used in the following 24 h after collection. For the experiments performed in the field, 70 individuals were selected and studied. A total of 137 individuals were used for the study.

### Leaf reddening and changes in leaf angle

Changes in abaxial colour (from green to red) and petiole angle of *S. hirsuta* leaves were studied in the field under natural conditions during autumn 2012. To study the leaf movement, 20 plants (and two leaves from each plant) were selected and marked. The angle between the petiole and the absolute horizontal was measured in the same two leaves from each plant with an inclinometer in October and in December 2012. To evaluate the influence of light on the leaf reddening process, canopy openness was measured over 28 *S. hirsuta* plants at the time of leaf fall of beeches (the main tree species of the study site) on 19 November 2012. The percentage of solar radiation reaching each leaf was determined by a digital camera, Nikon Coolpix 4500 equipped with a Nikon Fisheye Converted FC-E8, above each leaf. Hemispherical images were analysed with Gap Light Analyzer (GLA) Version 2.0 to transform image pixel intensities into sky or non-sky classes and to determine canopy openness. The same 28 leaves were collected and their abaxial side photographed in order to determine the extent of leaf reddening. All photographs were taken under the same photographic conditions (same lighting, distance to the camera, camera position, sensitivity, aperture and shutter speed). Images were analysed with ImageJ software for the quantification of the abaxial reddened area. The anthocyanic nature of leaf redness was confirmed as described in Esteban *et al*. (2008). Additionally, leaf sections of anthocyanic leaves were cut from fresh material using a razor blade, and mounted on a Nikon optical microscope (Nikon Eclipse E200, Japan) to determine the tissular location of the anthocyanins inside the leaves.

### Spectral scans of *S. hirsuta* leaves

Optical properties of leaf tissues were determined in anthocyanic and acyanic leaves of *S. hirsuta* collected in November and December (when differences in the reddening process among leaves allow one to find both kinds of leaves coexisting during few weeks) and immediately measured in the laboratory. Taking advantage of its easily removable epidermis, the transmittance and reflectance of photosynthetic photon flux density (PPFD) were directly measured in (i) whole anthocyanic leaves, (ii) isolated red abaxial epidermis, (iii) anthocyanic leaves without abaxial epidermis (it was removed) and (iv) whole acyanic leaves. Spectral scans were obtained using a spectroradiometer (UNISPECTM, FieldSpec UV/NIR portable spectral system, PP Systems, Amesbury, USA) with the optic fibre, leaf-clip holder and reference provided by the manufacturer. For the estimation of total transmittance and reflectance, a USB4000 with a HL-2000 Halogen Light Source, optic fibre and an ISP-30-6-R Integrating Sphere (Ocean Optics, Inc. World Headquarters, Dunedin, FL, USA) were used. To calculate the potential back-scattering coefficient, we multiplied the amount of light transmitted through the mesophyll at a given wavelength (based on the mesophyll transmittance value at each wavelength) by the reflectance of the abaxial epidermis at the same wavelengths.

### Light treatments and chlorophyll fluorescence measurements

It has been stated by Hughes *et al*. that the absorption of green light by abaxial anthocyanins may avoid internal scattering with the consequent reduction in the risk of photoinhibition (Hughes *et al*. 2008). To evaluate if abaxial anthocyanins confer any photoprotective advantage to *S. hirsuta* leaves, changes in the photochemical efficiency of the lower mesophyll were compared between anthocyanic and acyanic leaves after exposition to monochromatic blue, green or red light. Before light exposure treatment, chlorophyll (Chl) *a* fluorescence was measured in the abaxial surface of 10 randomly selected leaves of each type (anthocyanic and acyanic). The leaves had been dark-adapted for a minimum period of 30 min to allow the complete relaxation or oxidation of reaction centres in order to determine basal fluorescence (*F*_0_) using a Chl fluorometer PAM 2500 (Walz, Germany). A saturation pulse of 8000 µmol photons m^−2^ s^−1^ was applied to determine the maximal fluorescence (*F*_m_). The maximal photochemical efficiency of PSII was estimated by the ratio *F*_v_/*F*_m_ = (*F*_m_ − *F*_0_)/*F*_m_ (Genty *et al*. 1989). After *F*_v_/*F*_m_ measurements, five excised leaves of each type were exposed for 30 min to 200 µmol m^−2^ s^−1^ of blue (*λ* = 465–475 nm), green (*λ* = 515–525 nm) or red (*λ* = 620–630 nm) light provided by 6 W LED lamps (Clover Led, Spain). Samples were illuminated from the adaxial side of the leaves (the side from which they naturally receive light in the field). After 30 min of exposure to the corresponding monochromatic light, the actual photochemical efficiency of PSII under illumination, the so-called quantum yield of PSII (ΦPSII), was measured and estimated as (*F*_m′_ − *F*_t′_)/*F*_m′_ (Genty *et al*. 1989) for the abaxial surface of light-adapted leaves. Chl fluorescence was measured in the abaxial side to obtain information on the photosynthetic performance of the mesophyll cells of the lowest layers, which are closest to the anthocyanic abaxial epidermis. The differences in the efficiency of the use of light between the two kinds of leaves were estimated by the different percentages of decrease observed between *F*_v_/*F*_m_ and ΦPSII (percentage of decrease in photochemical efficiency = [(*F*_v_/*F*_m_ − ΦPSII)/(*F*_v_/*F*_m_)] × 100). Afterwards, the same leaves were incubated for 30 min in darkness and the *F*_v_/*F*_m_ measured for both the abaxial and the adaxial sides of each leaf.

### Germination tests

**Figure 1.**
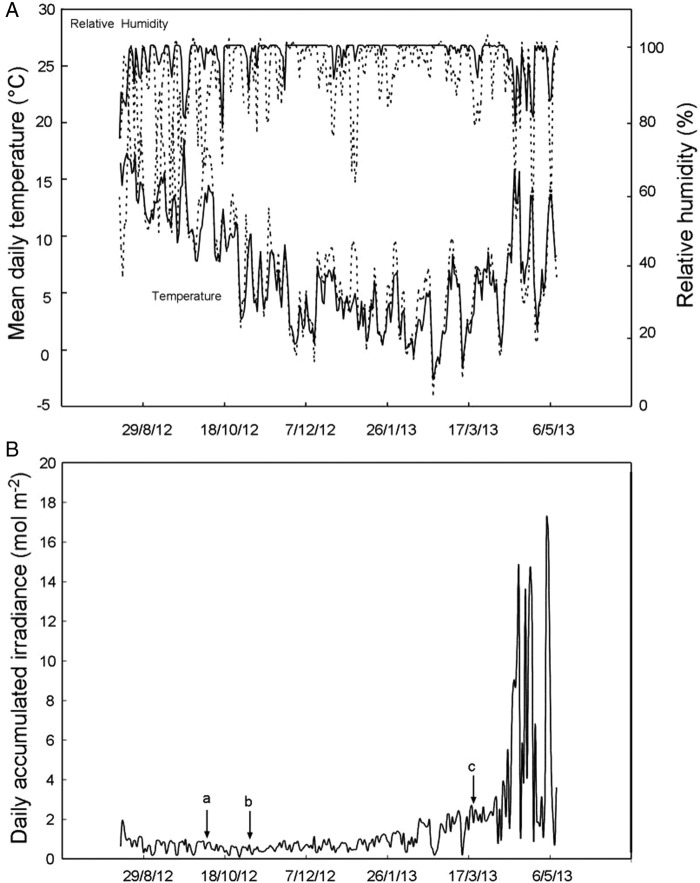
Environmental characterization and meteorological conditions of the habitat of *S. hirsuta* in Monte Santiago along the course of the year (data depicted from summer 2012 to spring 2013). In (A), daily mean temperature and RH within the forest (solid lines), and in a reference site located out of the forest (dashed lines) are shown. In (B), daily accumulated irradiance is depicted. At point a, the overstorey still had green leaves; at point b, overstorey leaves were browning; and at point c, the overstorey was without leaves.

The influence of anthocyanic leaves on the germinability of lettuce (*Lactuca sativa* var. Romaine) seeds was double checked under controlled conditions (greenhouse, at ∼20 °C) and under natural winter conditions (in the field, at −5–10 °C, see Fig. 1A). In the greenhouse, lettuce seeds (10 per well) were introduced, over wet filter paper, in multi-well plates (well dimensions: 16 mm Ø, 20 mm depth). Each well was covered with either acyanic (*n* = 12 different plants) or anthocyanic excised leaves (*n* = 13 different plants) of *S. hirsuta* (a total of 25 leaves were used). The sides of the plates were laterally covered by black film (to avoid lateral illumination) and finally closed with a transparent cover and left for 1 week under greenhouse conditions. In the field experiment, lettuce seeds were put over wet filter paper into small transparent capsules of 8 mm Ø and 5 mm depth (five seeds in each) and covered with a transparent and air-permeable film. Capsules with seeds were inserted in the top layer of soil below either acyanic (*n* = 10 different plants) or anthocyanic intact leaves (*n* = 9 different plants) of *S. hirsuta* (a total of 33 leaves were used) and were left for 1 month under its natural conditions. In both experiments, either wells or capsules had room enough for all the seeds to have exposure to the same amount of air and light (seeds were covering the surface of the wells and capsules without being piled on top of each other).

### Total nitrogen content and photosynthetic pigments analyses

To quantify total nitrogen content, 10 acyanic leaves and 10 anthocyanic leaves were collected in the field (December). For each leaf type, five replicates (two leaves each) were frozen in liquid nitrogen. Frozen samples were powdered with a manual mortar and then dried in an oven (60 °C for 48 h). The elemental analysis was performed with an EA-3000 CHNS analyser from EuroVector s.p.a. (Italy). Weighing was performed with a Sartorius SE2 microbalance (Germany) (elemental analysis). One to 5 mg of sample (or standard) measured with an accuracy of 0.001 mg were packed in a tin capsule and then introduced into the elemental analyser autosampler. Then the probe was burned in an automatic mode, and the resulting gaseous products were analysed chromatographically. The chromatogram obtained was processed using an original computer program Callidus 5.1. The calibration with respect to the reference sample was carried out for each series of analysis. Photosynthetic pigments and α-tocopherol were analysed in anthocyanic and acyanic leaves collected in the field in December 2013 (five leaves each) and measured by HPLC following the method of García-Plazaola and Becerril (1999, 2001). For the identification of the uncommon lactucaxanthin carotenoid, extracts of lettuce (which contains considerable amounts) were used as the reference.

### Statistics

One-way analysis of variance (ANOVA) was used to check for differences in (i) photosynthetic pigments and nitrogen content between anthocyanic and acyanic leaves, (ii) photochemical efficiency among different types of leaves subjected to monochromatic light and (iii) the percentage of seeds that germinated when placed below anthocyanic or acyanic leaves. The Kolmogorov–Smirnov test was used to check for data normality and Levene’s test to check for homogeneity of variances. Two-way ANOVA was additionally performed to test for differences in the percentage of decrease in photochemical efficiency among different leaves. ‘Monochromatic light’ and ‘anthocyanin effect’ were used as fixed factors. Student–Newman–Keul tests were used to discriminate among different treatments after significant *F*-test, and Cochran's test to test for heterogeneity of variances. Significant differences were assumed at *P* < 0.05. All analyses were performed using the SPSS 19.0 statistical package.

## Results

### Abiotic environment of *S. hirsuta*

Average climatic conditions of the Monte Santiago site, and the microclimate within the forest sites where *S. hirsuta* grows are depicted in Fig. 1. Overall, the data correspond with a temperate humid climate in which frequent fog events and cloudy days are responsible for a high mean RH (≥80 %) throughout the year (Fig. 1A). Extremes in daily mean temperatures were attenuated within the forest. Thus, during the study, daily mean temperatures ranged from a maximum of 25.9 to a minimum of −4 °C in the open field and between 18.3 and −2.2 °C in the forest understorey (Fig. 1A). Changes in RH were also attenuated under the beech canopy. The intensity of the irradiance that reaches the understorey is mainly determined by the properties of the overstorey and by the inclination of the sun in the sky. The highest light intensity was received in the understorey during early spring (Fig. 1B, beyond point *c*) when the intensity of sunlight increased and overstorey leaves had not sprouted yet, reaching the highest irradiation during the first days of May, immediately before beech budburst. This dramatic change in light conditions of the understorey together with the still low temperatures (Fig. 1A) makes early spring the most likely period for photoinhibitory events to occur.

### Characterization of leaf reddening process

**Figure 2.**
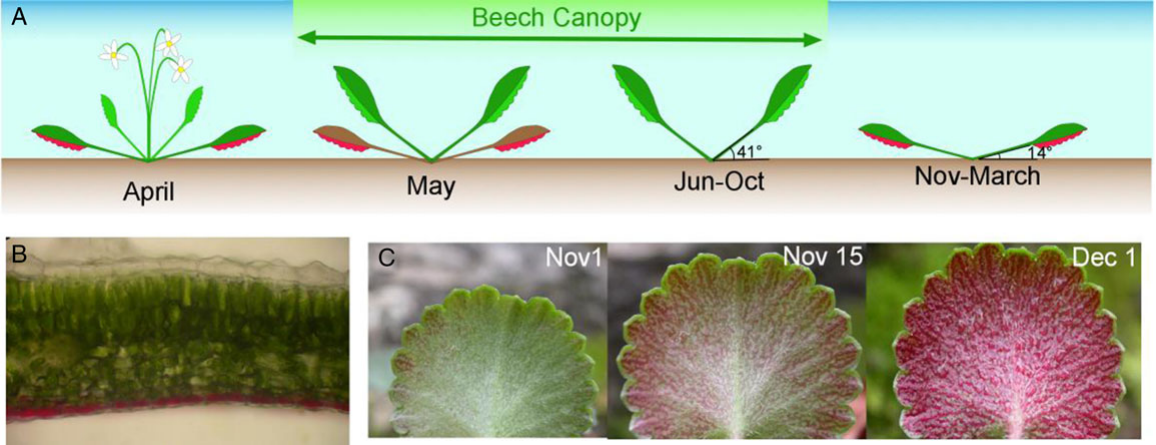
Characterization of anthocyanic leaves of *S. hirsuta*. In (A), the growth, seasonal change in leaf angle and reddening processes are represented. In (B), an optical microscopic image of anthocyanic leaves shows the abaxial anthocyanic epidermis responsible for the red colour. In (C) the reddening process of *S. hirsuta* leaves is depicted.

**Figure 3.**
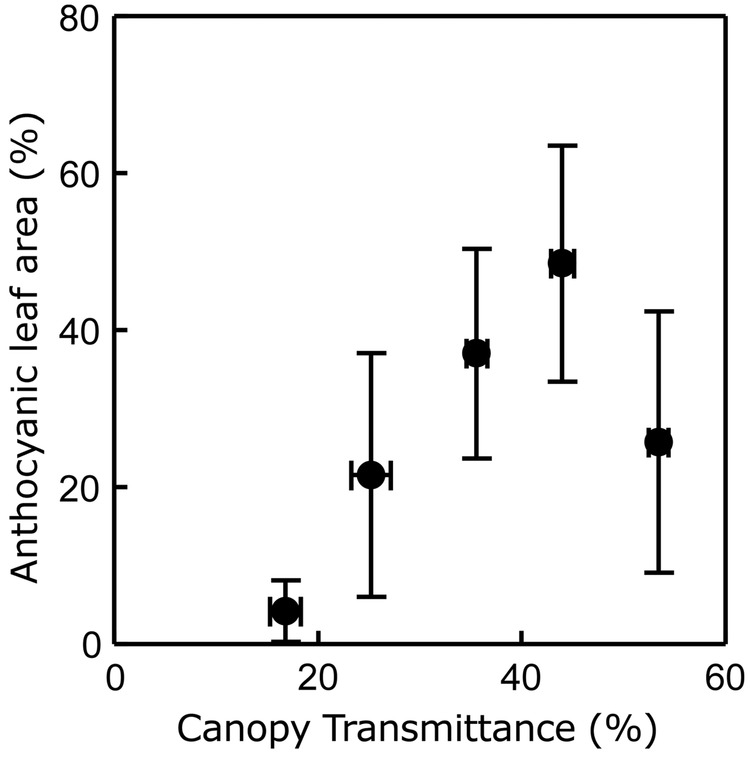
Influence of canopy openness on the winter reddening process of *S. hirsuta* leaves at the beginning of the winter. Each point represents the mean ± SE (*n* ≥ 4). The mean data of canopy openness and red area were grouped following steps of 10 % canopy openness over the whole canopy openness range, as follows: canopy openness ≤10, 11–20, 21–30, 31–40, 41–50, >51 %. All data were taken on a single day (19 November 2013).

Plants of *S. hirsuta* with coexistent acyanic and anthocyanic leaves naturally occur in the field under the same light environment (J.I. García-Plazaola, F. Míguez, R. Esteban, B. Fernández-Marín, pers. obs.). To identify the phenological changes of this trait, the developmental and seasonal behaviour of *S. hirsuta* leaves were studied (Fig. 2). The new leaves of *S. hirsuta* developed in spring (April), ∼1 month before the sprout of the beech overstorey (Fig. 2A). These new leaves were completely green and developed forming a steep angle (close to 90 °C) with respect to the substrate (Fig. 2A, April). The petiole angle decreased along the spring to a value close to 40° that was maintained during the rest of the growing season (Fig. 2A, May). Young acyanic leaves coexisted with old (senescent) anthocyanic leaves for several weeks until the latter started senescence at late spring (Fig. 2A, May). Curiously, during autumn the angle of the leaves was progressively reduced (mean leaf angle decreased from 41° in summer to 14° in winter) in parallel with the progressive accumulation of anthocyanins in the lower epidermal layer of cells (Fig. 2B and C). Leaves reached their definitive horizontal position in December (Fig. 2A). This matched temporally the leaf fall of the overstorey during autumn. Indeed, although all plants were abaxially red in winter, a positive relationship was observed on 19 November between the amount of light reaching the leaves of *S. hirsuta* and their state of the reddening process (Fig. 3). At the end of autumn (when anthocyanic leaves coexist with some still acyanic leaves due to the individual differences among plants with regard to the onset and speed of the reddening process), anthocyanic leaves showed higher levels of α-Tocopherol and AZ/VAZ and lower levels of total nitrogen content than acyanic leaves (Table 1). Both types of leaves, however, showed the same total Chl content (Table 1). Interestingly, the leaves of *S. hirsuta* possess an unusual photosynthetic pigment: lactucaxanthin, which was present at higher concentrations in acyanic leaves.

**Table 1.** Photosynthetic pigments and total nitrogen content of anthocyanic and still acyanic leaves of the same age (collected in December). Neoxanthin (N), lutein (L), lactucaxanthin (Lac), xanthophyll cycle pigments (VAZ), β-carotene (β-Car) and α-tocopherol (α-Toc) are expressed in mmol mol^−1^ Chl. Total Chl is expressed in μmol m^−2^, and the de-epoxidation index of xanthophyll cycle (AZ/VAZ) in relative units. Total nitrogen content is expressed in % of leaf DW. Values are mean ± SE (*n* = 5). Asterisks and bold *P* values denote significant differences in content among anthocyanic and acyanic leaves (*P* < 0.05).

Type of leaf	N	L	Lac*	VAZ	AZ/VAZ	β-Car	α-Toc	Ch a+b	Chl a/b	Nitrogen*
Anthocyanic	46.2 ± 1.2	92.3 ± 2.9	37.7 ± 1.6	59.1 ± 2.8	0.293 ± 0.028	79.0 ± 3.0	42.1 ± 5.4	500.5 ± 38.2	2.5 ± 0.1	1.73 ± 0.1
Acyanic	46.0 ± 0.4	86.7 ± 0.9	47.0 ± 0.8	54.0 ± 0.9	0.200 ± 0.043	76.1 ± 1.9	27.4 ± 4.2	516.7 ± 53.3	2.6 ± 0.1	2.39 ± 0.1
One-way ANOVA										
df	1	1	1	1	1	1	1	1	1	1
Mean^2^	0.122	78.261	216.743	64.974	0.022	21.005	541.717	657.722	0.013	1.089
*F*	0.030	3.404	27.304	2.944	3.309	0.653	4.656	0.061	0.880	11.440
*P*	0.868	0.102	**0.001**	0.125	0.106	0.443	0.063	0.811	0.376	**0.010**

### Effect of abaxial anthocyanin on light transmission through the leaf

**Figure 4.**
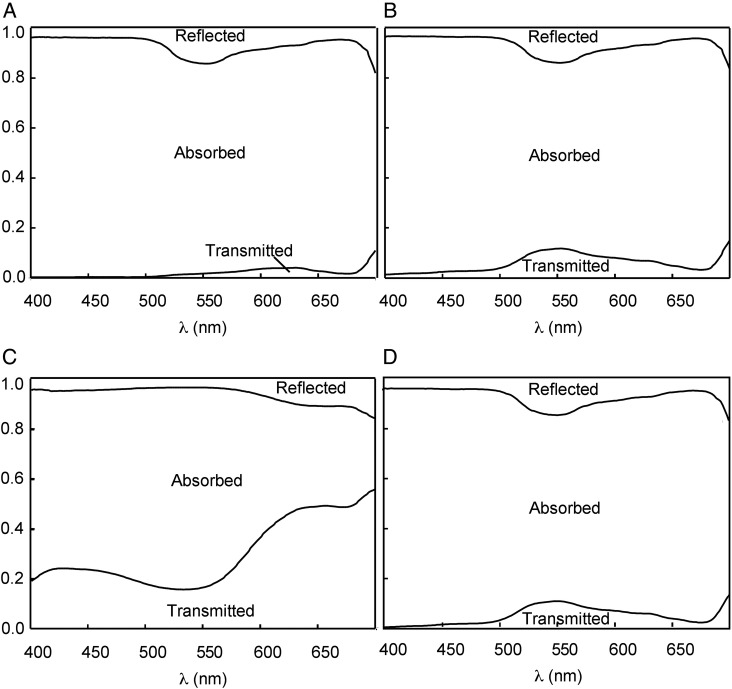
Optical properties (proportion of transmitted, reflected and absorbed PPFD) of anthocyanic leaves of *S. hirsuta*: intact leaf (A), mesophyll + adaxial epidermis (leaf in which the abaxial anthocyanic epidermis had been removed) (B), abaxial anthocyanic epidermis (C), and of intact acyanic leaf (D) are shown. The presence of chloroplasts in the stomata of the abaxial anthocyanic epidermis explains the absorption ∼650 nm (C).

Taking advantage of the easily removable lower epidermis of *S. hirsuta* leaves, the influence of abaxial anthocyanins on light distribution within the leaf profile was analysed by measuring spectral properties of (i) intact anthocyanic leaves (Fig. 4A), (ii) leaves with the abaxial anthocyanic epidermis removed (Fig. 4B), (iii) exscinded anthocyanic abaxial epidermis (Fig. 4C) and (iv) intact acyanic leaves (Fig. 4D). Light transmission through the leaves with removed anthocyanic epidermis was similar to that of entire acyanic leaves, showing an efficient absorption of all the PPFD with the exception of the green region of the spectrum (*λ* = 500–600 nm), in which they showed a higher proportion of transmitted light (Table 2). Coinciding with that region, the exscinded anthocyanic epidermis of the leaves showed a maximum of absorption at a *λ* ∼550 nm (Fig. 4C). This explains the very low PPFD transmittance of intact anthocyanic leaves in the green region (1.1 %, Table 2), compared with anthocyanic leaves lacking lower epidermis (6.6 %, Table 2) or with intact acyanic leaves (6.9 %, Table 2). Acyanic and anthocyanic leaves, when the abaxial anthocyanic epidermis was removed in the latter, show similar PPFD transmittance and spectral properties (Fig. 4 and Table 2). Assuming that the optical properties of excised epidermis are the same in an entire leaf, the back-scattering effect of a red epidermis was theoretically estimated to be 0.54 % of the total red radiation (600–700 nm; see Methods for theoretical back-scattering calculation).

**Table 2.** Total transmittance (%) in the green (500–600 nm) and red (600–700 nm) regions of the spectrum passing (i) an intact anthocyanic leaf, (ii) an acyanic leaf, (iii) mesophyll + adaxial epidermis of an anthocyanic leaf in which the abaxial anthocyanic epidermis had been removed (referred to as ‘mesophyll’) and (iv) an abaxial anthocyanic epidermis. For the latter, both transmittance and reflectance are shown, in order to estimate potential back-scattering. Means ± SE are shown (*n* = 5).

Range	Anthocyanic leaf transmittance	Acyanic leaf transmittance	Mesophyll transmittance	Anthocyanic epidermis transmittance	Anthocyanic epidermis reflectance
Green (500–600 nm)	1.1 ± 0.5	6.9 ± 0.9	6.6 ± 1.4	19.1 ± 2.5	13.3 ± 0.2
Red (600–700 nm)	2.5 ± 0.7	3.1 ± 0.6	3.2 ± 0.9	56.2 ± 2.8	17.1 ± 0.7

### Possible roles of abaxial anthocyanins: photoprotection and effects on seed germination

As abaxial anthocyanins interfered in the spectral pattern of light crossing the leaves of *S. hirsuta*, their possible role in photoprotection (attenuating internally scattered light during sunflecks and sun patches) was checked. For this purpose, the difference between the maximal and the actual photochemical efficiency (*F*_v_/*F*_m_ in the dark and ΦPSII after exposure to monochromatic red, blue or green light, respectively) was assessed as the % of decrease (see Methods for details) and compared between acyanic and anthocyanic leaves. As illustrated in Table 3, red light induced a comparable decrease (7.2–9.2 %) in their photochemical efficiency of the two types of leaves (no significant differences were found; see also Table 4). Green light induced the biggest decrease in the photochemical efficiency of the anthocyanic leaf (14.9 % vs 11.3–11 % of the acyanic leaf) although no significant differences were found. Under blue light, however, anthocyanic leaves showed a significantly larger decrease in their photochemical efficiency than that showed by acyanic leaves (the decrease was 22.6 % in the anthocyanic leaf, while it was 9.2 in the acyanic leaf, Table 3). The maximal photochemical efficiency was recovered in all treatments after 30 min of darkness **[see Supporting Information—Table S1]**.

**Table 3.** Decrease in the efficiency of light use (estimated as the percentage of decrease between *F*_v_/*F*_m_ and ΦPSII) in anthocyanic and acyanic leaves immediately after illumination with monochromatic light. Chl fluorescence was measured in the abaxial side of leaves before and after adaxial illumination with green, blue or red light as described in Methods. Values are means ± SE (*n* ≥ 4). Asterisks indicate significant difference among types of leaves (*P* < 0.05).

Leaves	Red	Green	Blue
	*λ* = 620–630 nm	*λ* = 515–525 nm	*λ* = 465–475 nm
Anthocyanic	9.2 ± 0.9	14.9 ± 2.8	22.6 ± 1.4*
Acyanic	7.2 ± 1.3	11.3 ± 2.6	9.2 ± 1.8

**Table 4.** Two-way ANOVA of the effects of monochromatic light (red, green and blue) and the presence of anthocyanins on the % of decrease in the photochemical efficiency of *S. hirsuta* leaves.

Source	Percentage of decrease in the photochemical efficiency			
	df	Mean^2^	*F*	*P*
Monochromatic light	2	111.16	8.26	0.003
Anthocyanic effect	1	229.31	17.05	0.001
Light × anthocyanic	2	74.45	5.53	0.014
Residual	17	13.45		

**Figure 5.**
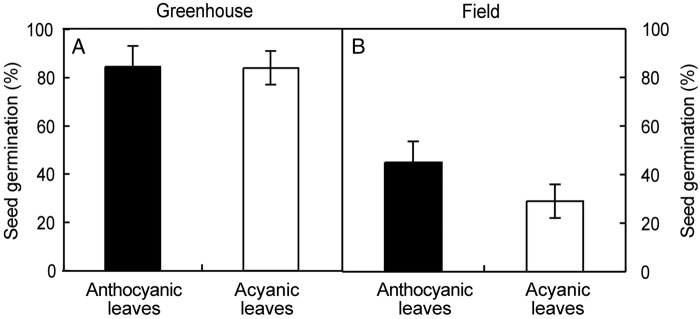
Effect of light filtering by anthocyanic leaves of *S. hirsuta* on the germination of lettuce seeds. The experiment was performed twice: under exscinded leaves in greenhouse conditions (A) or under living plants in natural conditions in the field (B). Bars represent the mean ± SE (*n* ≥ 12). No significant differences were found between anthocyanic and acyanic leaves in the number of seeds germinated underneath in any of the two experiments (*P* > 0.05). Details of the ANOVA analysis for (A): df = 1, mean^2^ = 2.186, *F* = 0.011, *P* = 0.917; for (B): df = 1, mean^2^ = 1943.29, *F* = 2.0 and *P* = 0.167.

As the photoprotective hypothesis was not supported by these results, we also explored whether changes induced by anthocyanins in the light reaching the underneath substrate (Fig. 4) may have effects on other below-growing plants. More specifically, we checked if this qualitative change together with the reduction in total light transmittance induced by abaxial anthocyanins of *S. hirsuta* may have an effect on seed germination of other species, as a mechanism to avoid competition in a light-limited environment (Fig. 5). When seeds of lettuce were sown below anthocyanic and below acyanic leaves of *S. hirsuta* in greenhouse conditions, no difference was found in the percentage of germination below both kinds of leaves (Fig. 5A). Similar results were found when seeds were sown below plants growing under natural conditions in the field (Fig. 5B). In this case, however, the percentage of germination was significantly lower than in the greenhouse (∼40 vs 80 % in the greenhouse). Thus, anthocyanic leaves had no significant effect on the germination of lettuce seeds placed underneath.

## Discussion

The studied population of *S. hirsuta* inhabited a shady environment with a mean accumulated irradiance lower than 2 mol of photons per day and square metre for most of the year (Fig. 1). In such a limiting environment, only the efficient use of resources may ensure survival. The accumulation of anthocyanins occurred in the abaxial epidermis of leaves and started in autumn (Fig. 2B and C) coinciding with the leaf shedding of the overstorey (Fig. 1). Reddening corresponded with increases in canopy openness of the overstorey (Fig. 3) and so with direct incident radiation (only a very small number of localized spots of a few individuals growing in very deep shade, where direct sunlight never reaches the plants, i.e. in the entry of a cave, did not accumulate visible amounts of anthocyanins during the entire winter, data not shown). This is in agreement with the well-known requirement of light to trigger anthocyanin synthesis, repeatedly described in other species (Chalker-Scott 1999; Hughes 2011). In parallel with the abaxial reddening, mature leaves progressively laid down until they reached an almost completely horizontal position, very close to the substrate that was kept during winter (Fig. 2A). Considering all that, a photoprotective role for abaxial anthocyanins could be expected, as has been suggested for other species when exposed abaxial leaf surfaces are vulnerable to high-incident light (Drumm-Herrel and Mohr 1985; Sherwin and Farrant 1998; Hughes and Smith 2007). Indeed, a photoprotective role for anthocyanins in red-undersurfaced leaves has been proposed in some tropical (Hughes *et al*. 2008, 2014), and in one temperate understorey species (Hughes and Smith 2007).

Nevertheless, in the case of *S. hirsuta* a photoprotective function for the abaxial anthocyanins appeared to be unlikely after experiments with monochromatic light (Table 3). Anthocyanic leaves suffered a deeper decrease in photochemical efficiency when exposed to monochromatic blue light. Lower photochemical efficiency of anthocyanic leaves has also been described in other species such as *Erythronium dens-canis* (Esteban *et al*. 2008), *Cistus creticus* (Kytridis *et al*. 2008; Zeliou *et al*. 2009) and *Pistacia lentiscus* (Nikiforou and Manetas 2010). In some of the species with red-sensitive individuals, lower leaf nitrogen content has been proposed to be involved in the higher vulnerability of anthocyanic plants against excess of light (Kytridis *et al*. 2008; Nikiforou *et al*. 2011). In the case of *Pistacia*, for instance, lower nitrogen content was related to lower Rubisco content and CO_2_ assimilation. Accordingly, the reduced capacity of the carboxylation reactions to act as photosynthetic electron sinks may explain the corresponding loss of PSII photon trapping efficiency, which could not be fully alleviated by the screening effect of the accumulated anthocyanins (Nikiforou *et al*. 2011). A similar situation could be expected for *S. hirsuta* as its anthocyanic leaves presented lower nitrogen content than its acyanic leaves (Table 1).

Nevertheless, a photoprotective role for the abaxial anthocyanins of *S. hirsuta* was further discarded when the photoprotective demands of its leaves were assessed in detail. In some other species with photoprotective red-undersurfaced leaves, anthocyanins are synthesized precisely when the abaxial surfaces of the leaves are exposed to relatively high irradiance (Hughes and Smith 2007), a situation in which a photoprotective demand could become more evident. Yet, the abaxial surfaces of anthocyanic leaves of *S. hirsuta*, are usually not exposed to direct light (Fig. 2A). In contrast, the accumulation of anthocyanins in autumn occurs in parallel with the repositioning of leaves to a horizontal position (14 °C, Fig. 2A), which enhances light capture (Niinemets 2010). Furthermore, the maximum irradiance in the understorey is in spring (April–May, Fig. 1B), when overstorey leaves had not yet been sprouted and when the sun angle was increasing in the dome of the sky. In that season (spring), in which maximal photoprotective demand should be required (particularly at the beginning of the season when increasing irradiance co-occurred with still low temperatures, Fig. 1), the new leaves of *S. hirsuta* emerge green with no visual content of anthocyanin, and vertical, with the abaxial side of the leaf exposed to the light (Fig. 2A). Thus, if abaxial anthocyanins of *S. hirsuta* could play a photoprotective function, why do young leaves (which are more exposed to potentially harmful irradiances) lack abaxial reddening?

Perennial herbs inhabiting the understorey of temperate deciduous forests present different strategies to face the winter. Some species, as *Viola hondoensis*, produce and shed leaves twice every year: winter leaves are produced in autumn and shed in spring when new leaves are produced. This succession of leaf generations seems to lead to a maximization of whole-plant photosynthesis by avoiding shelf-shading within the individual between new and old leaves (Hikosaka *et al*. 2010). This period (autumn to spring) coincides with the presence of anthocyanic leaves in *S. hirsuta*. Anthocyanins have been proposed to act as osmorregulators that could maintain cell turgor under circumstances that directly or indirectly may induce drought stress such as cold temperatures (Chalker-Scott 1999). This function, however, could be discarded in *S. hirsuta* leaves where anthocyanins are accumulated in the lower epidermis only. Furthermore, an osmorregulative function for anthocyanins has been considered unlikely by other authors (Manetas 2006; Hughes *et al*. 2013).

One emerging view of the role of red colouration in leaves proposes that it is frequently related with the biotic interactions with other organisms (Archetti *et al*. 2009 and references therein). This could be plausible for *S. hirsuta*, mainly considering that the photoprotective or light-collection hypotheses seem unlikely. In that sense, one remarkable effect that the lower epidermal anthocyanins of *S. hirsuta* have on its leaves is the change in the intensity and the quality of the light transmitted through the leaf, so that, an almost completely dark environment (total PPFD transmittance 1.8 %, Fig. 4 and Table 2) is generated below. Besides, anthocyanic leaves show a horizontal position, and remain close to the soil surface (Fig. 2A), further impeding the side illumination of the substrate underneath. Since light is required for seed germination of many species and most of them are in addition sensitive to spectral composition, especially to the ratio Red : Far Red (R : FR; 660 : 730 nm; Bewley and Black 1994), it would be then reasonable to suspect that abaxial anthocyanins may play a role in avoidance of germination of seeds from other potential competitor plants. Particularly, temperate forest herbs with small seeds (similar to those cohabiting with *S. hirstuta*) seem to need a high R : FR ratio (which signals high-light levels due to the absence of over-topping vegetation or leaf litter) to germinate (Jankowska-Blaszczuk and Daws 2007). While within the range of R : FR ratio values seed germination becomes generally possible over 0.5 (Vazquez-Yanes *et al*. 1990), the R : FR ratio of acyanic leaves was 0.05 and of anthocyanic leaves was virtually zero at 0.0034 (value, extracted from Fig. 4). Anthocyanic leaves of *S. hirsuta*, however, failed to prevent the germination of lettuce seeds, either under greenhouse or under field conditions (Fig. 5) even when it is widely known that red light is required for lettuce seed germination, and that FR prevents it (i.e. Borthwick *et al*. 1954).

Interestingly, several close relatives of this species (*S. umbrosa*, *S. tridactyles* and *S. rotundifolia*) were studied by Darwin (1875) as potential carnivore species, possessing glandular hairs able to trap small insects. Unfortunately, after Darwin, carnivorism has been neither confirmed nor rejected in this genus. *Saxifraga hirsuta* also contains glandular hairs, and insects are sometimes (in <2 % of the leaves, J.I. García-Plazaola, F. Míguez, R. Esteban, B. Fernández-Marín, pers. observations) found attached to them **[see Supporting Information—Fig. S1]**. It could be then hypothesized that abaxial redness might eventually attract insects, as has been demonstrated for some carnivorous species (Schaefer and Ruxton 2008). Nevertheless, the habitat of *S. hirsuta* does not correspond with the locations preferred by classic carnivorous plants, and the frequency of leaves with trapped insects is too low (3/125, data not shown) to justify a carnivorous habit in this species. Furthermore, insects were found both in anthocyanic and in acyanic leaves **[see Supporting Information—Fig. S1F]**, further discarding a function of anthocyanins in the attraction of insects as prey.

## Conclusions

Overall, our results do not support a function for abaxial anthocyanins either as photoprotectants or as enhancers of light capture. A solid conclusion of the present study is that horizontal foliage position, together with the convex leaf shape, generates a semi-closed space beneath each leaf which receives <2 % of the, yet highly filtered, incident light reaching the forest understorey. Thus, the maximum PPFD reaching the soil below the *S. hirsuta* canopy is <0.2 µmol m^−2^ s^−1^, a value far below the compensation point of shade most tolerant species. Therefore, inhibition of vital processes of competitors could be a plausible explanation for the red abaxial colouration of *S. hirsuta*.

## Sources of Funding

We acknowledge post-doctoral grants from the Research Vicerrectorate of the UPV/EHU, Marie Curie IEF grant (328370 MELISSA) from the European FP7-327 PEOPLE, JAE-Doc fellow from the Spanish National Research Council (CSIC) and pre-doctoral fellowship from the Basque Government. The work was supported by the Spanish Ministry of Education and Science (BFU 2010-15021) and co-funded by Feder and by the Basque Government (UPV/EHU-GV IT-624-13)].

## Contributions by the Authors

J.I.G.-P. was the principal investigator and supervisor of F.M., V.C., B.F.-M. and R.E. J.I.G.-P. and R.E. performed field measurements of leaf angles. R.E. performed photoinhibition experiments. V.C. and U.A. performed image analysis. U.A. prepared nitrogen analyses. F.M. and B.F.-M. performed the germination tests and the canopy measurements. J.I.G.-P. and M.P.-M. performed the spectral analyses. J.I.G.-P. and B.F.-M. drafted the manuscript. R.E. and J.M.B. analysed the meteorological data. B.F.-M., R.E. and J.M.B. performed the statistical analyses. All authors contributed to the final version of the manuscript.

## Conflict of Interest Statement

None declared.

## Supporting Information

The following additional information is available in the online version of this article –

**Figure S1.** Possible indices of carnivorism in *S. hirsuta* leaves. (A) Abaxially acyanic leaf. (B) The same leaf during the winter reddening process showing a dead attached insect (detail of the insect in C). (D) Cross-section of a leaf under optical microscope showing abaxial and adaxial hairs. (E) Details of the tip of a hair showing a dark content. (F) Attached insect in the abaxial side of an acyanic leaf.

**Table S1.** Maximal photochemical efficiency (*F*_v_/*F*_m_) of anthocyanic and acyanic leaves, before (*t*_0_) and after monochromatic light treatments. Measurements after light treatment were taken after 30 min of dark adaptation. *F*_v_/*F*_m_ values measured on the adaxial and the abaxial sides of the leaf are shown. No significant differences were found between leaf types or light treatments (*P* < 0.05). Details of the ANOVA for adaxial data: df = 7, mean square = 0.001, *F* = 1.624, *P* = 0.142. The Kolmogorov–Smirnov *Z*-test was used for heteroscedastic abaxial data, *P* > 0.05 in all comparisons.

Additional Information
