# Supplementary material for: Ecophysiological roles of abaxial anthocyanins in a perennial understorey herb from temperate deciduous forests
Source: AoB Plants. 2015 Apr 28;7:plv042. doi: 10.1093/aobpla/plv042 (PMC4481727; doi:10.1093/aobpla/plv042)
Supplement: Additional Information [file supp_7_plv042_index.html]

Ecophysiological roles of abaxial anthocyanins in a perennial understorey herb from temperate deciduous forests — Additional Information 

# Ecophysiological roles of abaxial anthocyanins in a perennial understorey herb from temperate deciduous forests

## Additional Information

Additional Information

- Supplementary Data - doc file
