# Supplementary material for: Ecophysiological roles of abaxial anthocyanins in a perennial understorey herb from temperate deciduous forests
Source: AoB Plants. 2015 Apr 28;7:plv042. doi: 10.1093/aobpla/plv042 (PMC4481727; doi:10.1093/aobpla/plv042)

###### Table S1 Maximal photochemical efficiency (Fv/Fm) of anthocyanic and acyanic leaves, before (t_0_) and after monochromatic light treatments. Measurements after light treatment were taken after 30 min of dark adaptation. Fv/Fm values measured on the adaxial and the abaxial sides of the leaf are shown. Values are means ± SE (n≥4 leaves). No significant differences were found between leaf types or light treatments (*P*<0.05). Details of the ANOVA for adaxial data: df= 7, mean square= 0.001, F= 1.624, p= 0.142. Kolmogorov-Smirnov Z-test was used for heterocidastic Abaxial data, P>0.05 in all comparisons.

| **Leaf side** | **Leaf type** | **t_0_** | **Red led** | **Blue led** | **Green led** |
| --- | --- | --- | --- | --- | --- |
| Adaxial | Anthocyanic | 0.736 ± 0.008 | 0.746 ± 0.007 | 0.73 ± 0.01 | 0.746 ± 0.007 |
|  | Acyanic | 0.759 ± 0.007 | 0.739 ± 0.005 | 0.735 ± 0.006 | 0.739 ± 0.005 |
| Abaxial | Anthocyanic | 0.697 ± 0.011 | 0.706 ± 0.014 | 0.709 ± 0.008 | 0.706 ± 0.014 |
|  | Acyanic | 0.722 ± 0.006 | 0.71 ± 0.007 | 0.713 ± 0.007 | 0.710 ± 0.007 |

###### Fig. S1 Possible indices of carnivorism in *S. hirsuta* leaves. A: abaxially acyanic leaf. B: the same leaf during the winter reddening process showing a dead attached insect (detail of the insect in C). D: cross section of a leaf at optical microscope showing abaxial and adaxial hairs. E: detail the tip of a hair showing a dark content. F: attached insect in the abaxial side of an acyanic leaf.


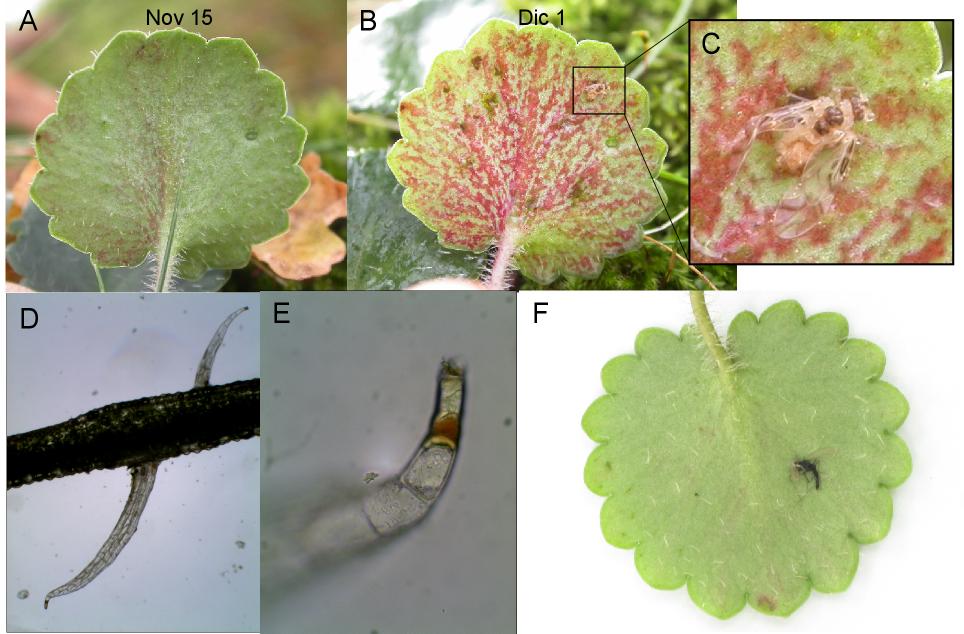

Supplement: Additional Information [file supp_plv042_plv042supp_Data.doc]
